# Evolving Landscape of Modern Contraceptive Use in Ethiopia: A Two-Decade Analysis

**DOI:** 10.3389/ijph.2025.1607680

**Published:** 2025-04-11

**Authors:** Ermias Tadesse Beyene, Sumin Kim, Seungman Cha, Yan Jin, Myunggu Jung

**Affiliations:** ^1^ Department of Global Development and Entrepreneurship, Graduate School of Global Development and Entrepreneurship, Handong Global University, Pohang, Republic of Korea; ^2^ Department of Microbiology, Dongguk University College of Medicine, Gyeongju, Republic of Korea; ^3^ Center for Global Development, Yonsei University Health System, Seoul, Republic of Korea

**Keywords:** family planning, reproductive women, trend, demographic and health surveys (EDHS), multilevel analysis

## Abstract

**Objective:**

The prevalence of modern contraceptive use in Ethiopia has increased in the past two decades. Despite these efforts, unmet needs for modern contraception persist, with limited knowledge on regional variations and determining factors.

**Method:**

We analyzed data from the Ethiopian Demographic and Health Surveys (EDHS) from 2000, 2005, 2011, 2016, and 2019. Descriptive statistics examined regional variations and trends in modern contraceptive use among married, non-pregnant women. Multilevel analysis identified individual and community-level factors influencing modern contraceptive use.

**Result:**

Nationally, modern contraceptive prevalence (mCP) increased, but regional disparities widened, notably between Addis Ababa and Somalia, from 34.8 to 51.8 percentage points. Factors such as community wealth, residence, age, education, and number of children significantly influenced contraceptive use. The greatest increase in mCP was among women aged 15–24, with a rise of 49.4 percentage points from 2000 to 2019.

**Conclusion:**

Tailored reproductive health services at both individual and community levels are essential to address the growing regional disparities in modern contraceptive use among married women in Ethiopia.

## Introduction

Family planning (FP) promotes maternal, newborn, and child health [1]. Providing access to FP can prevent maternal and child mortality associated with pregnancy and childbirth [2–4]. In 2019, of the 1.9 billion women of reproductive age worldwide, 1.1 billion required family planning. However, only 842 million of these women were using contraception [5, 6]. The Sustainable Development Goals (SDGs) aim for countries to achieve universal access to sexual and reproductive healthcare services, including Family Planning, information, and education, and to integrate reproductive health into National Strategies and Programs by 2030 [7].

Since 1997, Ethiopia’s health system has successfully implemented four phases of health sector development plans, followed by the Health Sector Transformation Plan (HSTP) which began in 2016. This plan established key impact metrics, including reducing teenage/adolescent pregnancy rates from 12% to 3%. According to the new National Reproductive Health Strategy (2016–2020), the goal of family planning is to reduce unintended pregnancies and enable individuals to achieve their desired family size [8]. Ethiopia’s 2030 Agenda and SDGs align with the United Nations Leaving No One Behind initiative [9, 10]. For the past two decades, Ethiopia has seen a rising trend in the use of modern contraceptives [11, 12]. Despite the increasing modern contraceptive prevalence (mCP) in Ethiopia, evidence indicates that the modern contraceptives use differs significantly by geography [12]. Numerous studies have shown that the modern contraceptives utilization is significantly associated with individual-level factors age [12–15], education [12, 14–22], wealth [12–19, 21, 22], employment status [13, 16–23], number of living children [13–15, 18, 20, 21, 23], exposure to media [14–16, 18, 20, 22, 24], experience of terminated pregnancy [16, 23], and religion [11, 15, 16]; and with the community-level factors such as place of residence [11–17, 21–23], and region [12, 13, 16].

Previous studies on mCP in Ethiopia have yielded varied results. For instance, regarding educational status, some studies have discovered that women who have completed secondary or higher education are more likely to use modern contraceptives than those without any education [12, 15, 16, 22]. On the other hand, some studies have found a weak association between the use of modern contraceptives among women and education [14–16]. Therefor this study aims at clarifying and expanding understanding of the factors influencing modern contraceptive use in Ethiopia. In addition, although several studies have focused on the factors of contraceptive use in a single specific year [12–16, 23], understanding the dynamics in the use of modern contraceptives over time can provide insights into how contraceptive use responds to changes in women’s individual- and community-level characteristics. As a result, our study aims to explore trends over time and determine whether the relationship between the various potential factors and contraceptive use has changed due to evolving social norms or healthcare access. Furthermore, while some studies have analyzed trends and factors in contraceptive use, no research has examined the use and determinants of contraceptives over an extended period of nearly two decades (from 2000 to 2019) in Ethiopia [1, 18].

Consequently, the objective of this study is to fill the research gap by analyzing the trends and determine the factors influencing the use of modern contraceptives among married and non-pregnant women aged 15–49 at both individual- and community-levels in Ethiopia from 2000 to 2019 and delineate the regional variation in modern contraceptive use.

## Methods

### Data Collection, and Data Source

**FIGURE 1:**
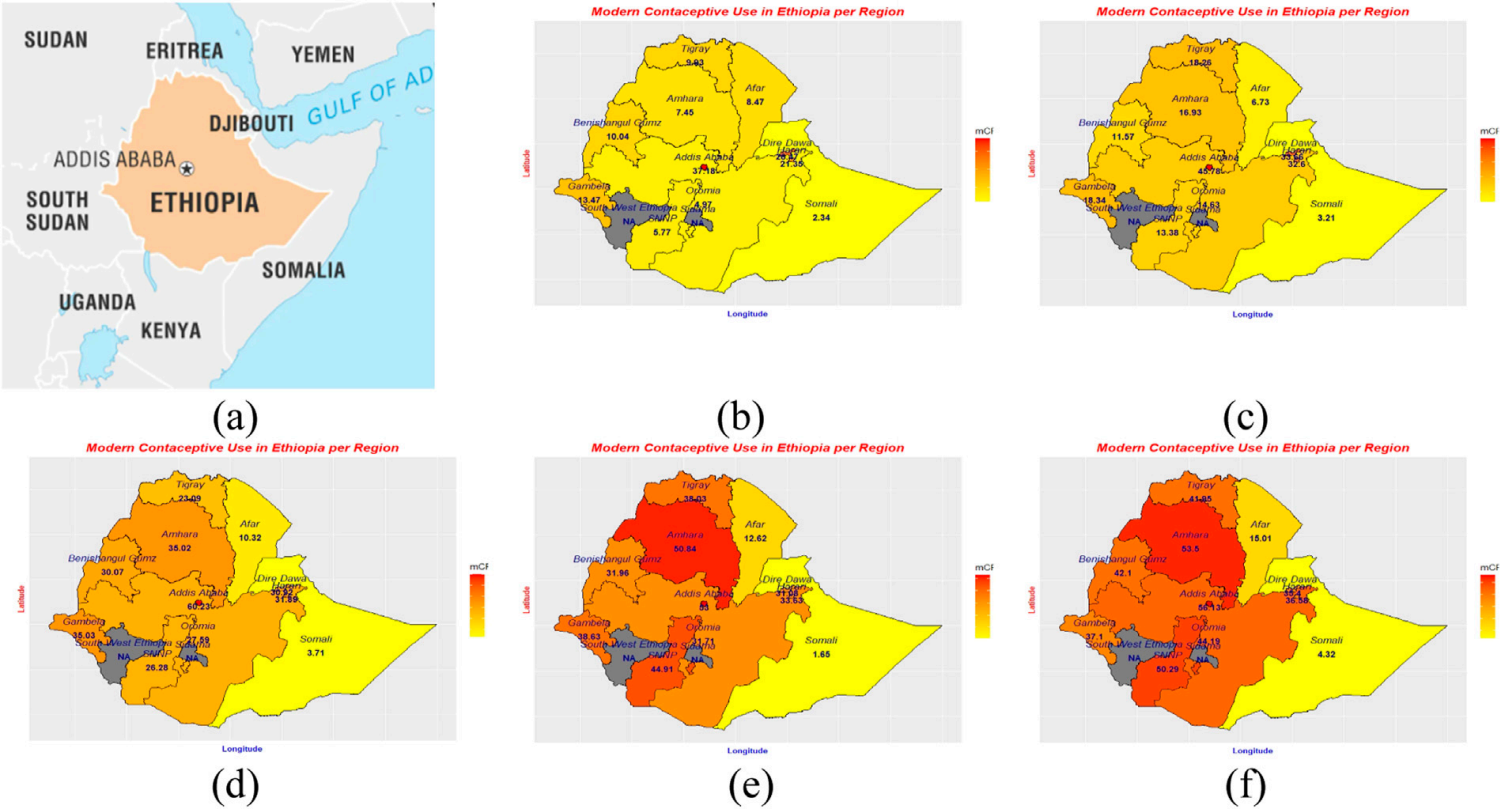
Location of Ethiopia **(A)** and modern contraceptive use by region 2000 **(B)**, 2005 **(C)**, 2011 **(D)**, 2016 **(E)**, 2019 **(F)**. Source: Britanica (https://www.britannica.com/), The DHS program.

For this study, the Ethiopia Demographic and Health Survey (EDHS) dataset of five consecutive survey years: 2000, 2005, 2011, 2016, and 2019 was utilized. Details of the sampling method and design can be found in the EDHS reports from the respective survey years [25–28]. Briefly, the selection of samples in the EDHS was conducted using a two-stage sampling procedure. In the initial stage enumeration areas (EAs) which are geographic areas containing appropriate numbers of residential units serving as counting units for the census were selected from the nine regions and two city administrations. Compared to women who are single, divorced, or bereaved women those who are married or in a union are highly likely to be sexually active. This is true in Ethiopia, where sex outside of marriage is uncommon [14]. Consequently, our analysis focused exclusively on married and non-pregnant women, as unmarried women were excluded due to the relatively uncommon occurrence of out-of-wedlock childbearing in Ethiopia [29]. The total sample size is depicted in Figure 1. Data cleaning was carried out using SPSS software version 24, while R software version 4.1.2 was used for the statistical analysis and graph plotting.

### Ethical Considerations

In addition to the granted government permission, the necessary ethical procedures involving confidentiality assurance and informed consent were followed by the Ethiopian Demographic and Health Surveys (EDHS). The DHS Program approved the secondary data analysis for this study.

### Study Variables

#### Outcome Variable

The variable Modern contraceptive use was used as our outcome of interest measured in our study. It was analyzed as a dichotomous variable (0 = yes, 1 = no) among the married and non-pregnant women of reproductive age. This variable was derived from the categorical variable “current use [of contraceptives] by type.”

#### Explanatory Variables

Literature review served for the selection of all our individual and the two community level variables (region and place of residence), but we aggregated the individual level variables by cluster to get the other community level variables. Region and place of residence are not variables that are unique to each individual rather they are group names assigned to a certain community. Additionally, we acknowledge that factors, such as exposure to media and visits to health facilities, may be influenced by broader community-level access factors such as electricity, road infrastructure, and proximity to health facilities. In our study, we classified variables based on their primary source of explanatory variation into two categories: those at the individual and those at the community-levels.

##### Individual-Level Variables

These were variables that reflect personal behaviors, choices, or characteristics of individuals or households, such as: respondents’ age, level of education, religious affiliation, household wealth index, the women’s employment status, the number of children in the family, any experience of terminated pregnancy (abortion), number of births in the last 3 years, exposure to FP messages through radio, TV, or newspapers, visits to health facilities in the past 12 months, husband’s desire for children, and husband’s or partner’s level of education.

##### Community-Level Variables

These included structural or contextual characteristics of the environment in which individuals reside. We used the two community variables, namely, region and place of residence, directly from the dataset. The community-level variables, including community-level work status, community-level wealth index, community-level media exposure, and community-level religion (percentage of Muslims), were derived from the EDHS data. To obtain these community-level variables, we first aggregated the corresponding individual-level variables within each cluster. For example, to create the community-level work status variable, we calculated the percentage of married and non-pregnant women who were employed in each cluster within the EDHS dataset. We then categorized this variable as ‘high’ or ‘low’ based on its value relative to the national-level percentage for each survey year. Similarly, for the community-level analysis of religion, we calculated the percentage of Muslims in each cluster relative to the national-level percentage of the dominant religion (Orthodox) for each survey year. Clusters were categorized as “high” (Muslim-dominant areas) if the percentage of Muslims in the cluster exceeded the national percentage of the Orthodox population; otherwise, they were categorized as “low.”

### Analysis

To address the disproportional distribution of the sample across various regions, including both urban and rural areas and to account for potential variation in response rates, sampling weights are utilized.

#### Regional Variation in Modern Contraceptive Use

To examine regional variation in modern contraceptive use, we employed descriptive statistics. This data is then visualized through tables, graphs, and maps to illustrate the variation and trend in modern contraceptive prevalence over times. To evaluate the result in greater detail, we divided the period broadly into two phases. The first phase, phase 1, spans from 2000 to 2011, while the second phase, phase 2, covers 2011 to 2019. Additionally, we depict the regional variation and the trend using a graph and maps.

#### Factors Associated With Modern Contraceptives Use at Individual- and Community-Levels

In this study, we employed a two-level mixed-effect logistic regression to identify the primary individual- and community-level factors determining the use of modern contraceptives among women. After running a two-level bivariate mixed-effect logistic regression we incorporated categorical variables with a p-value of less than 0.25 [13, 16] in the final multilevel mixed-effect logistic regression. We constructed four successive models: the intercept-only (null model), fixed effect model, random effect model, and mixed effect model. We evaluated their Akaike information criterion (AIC) and Bayesian information criterion (BIC) values to identify the model that best fit the data. The goodness of fit for the final adjusted model was estimated using AIC and BIC statistics and verified using a log-likelihood test in comparison to the preceding models. The lower AIC and BIC values the better model fit. The model we used for the analysis is as follows:

$$\text{Logit }\left(P_{\text{ij}}\right)=B_{00}+B_{10}*x_{\text{ij}}+B_{01}*X_{j}+U_{0j}+\varepsilon_{\text{ij}}$$

Where i and j are the individual (level 1) and cluster (level 2) units, respectively; Pij is the probability of modern contraceptive use for woman i in cluster j; *B*_00_ is the fixed intercept (a general constant term); *B*_10_ and *B*_01_ are the fixed coefficients; x and X represent the individual-level and the community-level explanatory variables respectively; *U* indicates the random effects for jth cluster; and ε shows unmeasured factors that may influence the primary outcome variable. We also tested the existence of any interaction among the explanatory variables. To evaluate individual variation, we conducted an analysis of the fixed effect using the adjusted OR, complemented by 95% CIs. For assessing community-level variation (random effect), we calculated the intraclass correlation coefficient (ICC) value.

#### Trends in Modern Contraceptive Use

We utilized descriptive statistics to illustrate the trends of the study variables. The results were presented in tables using frequencies and percentages. Additionally, graphs and narrative descriptions were employed to further depict the trend in modern contraceptives use.

## Results

### Background Characteristics of Married and Non-Pregnant Women

The analysis included a total of 36,969 married and non-pregnant women. The majority of these women fell within the 25–34 age group, accounting for 14,630 (39.7%), while the smallest group was aged 15–24, comprising 8,788 (23.3%) of the total. Significant proportion of the married and non-pregnant women had no education, 24,798 (68.8%), with only 1,242 (2.4%) having received higher education. In terms of religious affiliation, Orthodox women made up the largest group at 14,708 (44.5%), followed by Muslims at 15,015 (31.7%) and Protestants at 6,182 (21.0%). Women with 1-4 children represented about 22,052 (57.9%) of the total married and non-pregnant women, while those with five or more children constituted 11,483 (33.9%) of the total. Approximately 23,072 (59.6%) of the married and non-pregnant women had heard about FP through at least one media source. The background characteristics of the married, non-pregnant women are detailed in Table 1.

**TABLE 1:** Background characteristics of married non-pregnant women, of the Ethiopian Demographic and Health Survey years 2000, 2005, 2011, 2016, and 2019 (N = 36,969).

Variables	2000 (n = 7,953)		2005 (n = 7,400)		2011 (n = 8,265)		2016 (n = 8,492)		2019 (n = 4,859)		2000–2019 (N = 36,969)	
	Freq	%	Freq	%	Freq	%	Freq	%	Freq	%	Freq	%
Age												
15–24	1928	25.60	1804	23.93	1960	23.34	1973	20.87	1,123	22.80	8,788	23.31
25–34	2,960	35.86	2,884	38.99	3,311	40.24	3,469	43.03	2006	40.25	14,630	39.67
35–49	3,065	38.54	2,712	37.08	2,994	36.42	3,050	36.10	1730	36.94	13,551	37.02
Woman’s education level												
No education	6,286	83.20	5,477	78.50	5,477	67.14	4,991	62.72	2,567	52.67	24,798	68.85
Primary	985	11.66	1,111	15.05	2089	26.38	2,295	27.38	1,540	35.77	8,020	23.25
Secondary	608	4.56	686	5.43	415	3.52	745	5.96	455	7.88	2,909	5.47
Higher	74	0.58	126	1.02	284	2.96	461	3.94	297	3.68	1,242	2.44
Religion												
Orthodox	3,556	49.95	3,205	46.12	3,076	45.37	3,125	41.35	1746	39.48	14,708	44.45
Protestant	1,089	15.90	1,186	18.35	1,396	20.31	1,560	22.40	951	27.83	6,182	20.96
Muslim	2,952	29.62	2,763	31.96	3,577	31.94	3,650	33.83	2073	31.09	15,015	31.69
Other religion	356	4.53	243	3.57	212	2.38	157	2.43	89	1.6	1,057	2.90
Children												
None	826	9.20	642	7.35	756	8.22	761	7.17	449	9.36	3,434	8.26
1–4	4,782	58.69	4,427	57.56	4,891	58.02	5,037	57.64	2,915	57.51	22,052	57.88
≥5	2,345	32.11	2,331	35.08	2,618	33.76	2,694	35.19	1,495	33.13	11,483	33.85
Heard FP message via media												
No FP message	6,376	84.97	5,257	73.82	5,500	66.56	5,939	72.56	Na	Na	23,072	59.58
FP message	1,577	15.03	2,143	26.18	2,765	33.44	2,553	27.44	Na	Na	9,038	20.42
Husband’s education level												
No education	5,048	66.13	4,315	60.28	4,202	50.08	3,863	46.45	Na	Na	17,428	44.59
Primary	1,628	22.76	1722	26.86	2,811	38.82	2,647	36.87	Na	Na	8,808	25.06
Secondary	998	8.79	1,072	10.62	655	5.54	1,028	9.09	Na	Na	3,753	6.81
Higher	235	1.90	260	2.07	512	4.90	873	6.83	Na	Na	1880	3.14
Don’t know	35	0.43	13	0.17	77	0.66	81	0.76	Na	Na	206	0.40
Woman’s work status												
No	3,501	43.66	5,501	74.31	5,573	64.19	5,723	69.20	Na	Na	20,298	50.27
Yes	4,449	56.34	1898	25.69	2,685	35.81	2,769	30.80	Na	Na	11,801	29.73
Region												
Tigray	733	6.29	654	6.03	837	6.25	857	6.62	377	6.07	3,458	6.25
Afar	540	1.32	540	1.22	817	1.06	738	0.93	403	1.07	3,038	1.12
Amhara	1,145	27.22	1,148	26.68	1,181	28.99	1,011	24.31	580	23.58	5,065	26.16
Oromia	1,388	38.11	1,250	36.33	1,172	39.11	1,135	38.68	624	38.79	5,569	38.20
Somali	463	1.07	434	3.98	541	2.21	798	2.99	348	4.37	2,584	2.92
Benishangul-Gumuz	597	1.13	535	1.00	743	1.21	709	1.12	460	1.15	3,044	1.12
SNNPR	1,094	21.45	1,139	21.24	928	17.13	1,044	20.93	618	20.39	4,823	20.23
Gambela	554	0.30	435	0.35	606	0.37	626	0.29	380	0.42	2,601	0.35
Harari	407	0.22	411	0.24	499	0.26	486	0.24	373	0.26	2,176	0.24
Addis Ababa	590	2.50	473	2.50	489	3.09	577	3.40	314	3.31	2,443	2.96
Dire Dawa	442	0.39	381	0.43	452	0.31	511	0.49	382	0.57	2,168	0.44
Place of residence												
Urban	1,571	11.65	1,513	10.95	1863	17.62	2,136	16.05	1,318	27.36	8,401	16.73
Rural	6,382	88.35	5,887	89.05	6,402	82.38	6,356	83.95	3,541	72.64	28,568	83.27

Note: FP: family planning; SNNPR, southern nations, Nationalities, and Peoples’ Region; Other religion: Catholic, traditional religions, and other unspecified religions.

### Regional Variations in Modern Contraceptive Use

In the years 2000 and 2005, Addis Ababa led in the percentage of modern contraceptive use registering 21.4% and 32.6% respectively while Dire Dawa city administration and the Harari region follow closely behind. By 2011, Addis Ababa had the highest percentage (60.2%) in modern contraceptive use, followed by Gambela, and Amhara each with 35.0% and Harari a 31.9%. The regions of Addis Ababa, Amhara, and SNNPR held the first, second, and third ranks with a 53.0%, 50.8%, and 44.9% in 2016 and a 56.1%, 53.5%, and 50.3% in 2019 in terms of modern contraceptive use. Throughout the period from 2000 to 2019, the Somali region recorded the lowest percentage of modern contraceptive use registering a 2.3%, 3.2%, 3.7%, 1.7%, and a 4.3% respectively. The disparity between Addis Ababa and Somalia, the regions with the highest and lowest percentages of modern contraceptive use in subsequent survey years, was 34.8%p in 2000. Similarly, it was 42.6%p in 2005, 56.5%p in 2011, 51.3%p in 2016, and 51.8%p in 2019. However, nearly all regions demonstrated a consistent increase in the use of modern contraceptives over the past two decades, with the exception of the Somali region (Figures 1A–F; Table 2).

**TABLE 2:** Regional variation and trends in modern contraceptive use among married non-pregnant women aged 15–49 years by selected characteristics in 2000, 2005, 2011, 2016, and 2019 Ethiopian Demographic and Health Surveys (EDHS) (N = 36,969).

Characteristics	EDHS 2000	EDHS 2005	EDHS 2011	EDHS 2016	EDHS 2019	Percentage point difference		
						Phase 1 (2011–2000)	Phase 2 (2019–2011)	Total (2019–2000)
Age category								
15–24	5.2	15.3	33.4	43.8	54.7	28.2	21.3	49.5
25–34	9.2	17.5	34.0	44.0	52.9	24.8	18.9	43.7
35–49	6.5	13.6	22.4	30.9	32.4	15.9	10.0	25.9
Women’s education level								
No education	4.2	11.0	23.5	33.8	35.7	19.3	12.2	31.5
Primary	14.8	25.1	37.7	44.7	54.2	22.9	16.5	39.4
Secondary	35.7	48.9	57.3	59.5	62.9	21.6	5.6	27.2
Higher	51.0	44.2	63.2	55.6	69.6	12.2	6.3	18.6
Religion								
Orthodox	8.8	20.5	35.5	48.0	52.2	26.7	16.7	43.4
Protestant	5.2	14.0	30.6	46.8	52.8	25.4	22.2	47.6
Muslim	6.0	10.0	21.6	24.6	30.9	15.6	9.3	24.9
Other religion	3.5	9.2	16.9	24.4	50.0	13.4	33.1	46.5
Total number of children								
None (no child)	3.5	8.2	23.9	36.7	30.8	20.4	6.9	27.3
1–4	7.4	16.9	34.7	45.6	54.8	27.3	20.1	47.4
>=5	7.7	14.9	22.2	29.3	34.1	14.5	11.9	26.4
Heard FP message via media								
No	4.7	10.9	23.7	36.0	Na	19.0	Na	Na
Yes	21.1	28.7	41.4	47.8	Na	20.3	Na	Na
Women work status								
No	6.5	13.6	25.5	36.4	Na	19.0	Na	Na
Yes	7.7	21.0	36.9	45.5	Na	29.2	Na	Na
Region								
Tigray	9.9	18.3	23.1	38.0	41.9	13.2	18.8	32.0
Afar	8.5	6.7	10.3	12.6	15.0	1.8	4.7	6.5
Amhara	7.4	16.9	35.0	50.8	53.5	27.6	18.5	46.1
Oromia	5.0	14.6	27.6	31.7	44.2	22.6	16.6	39.2
Somali	2.3	3.2	3.7	1.7	4.3	1.4	0.6	2.0
Ben-Gumz	10.0	11.6	30.1	32.0	42.1	20.1	12.0	32.1
SNNPR	5.8	13.4	26.3	44.9	50.3	20.5	24.0	44.5
Gambela	13.5	18.3	35.0	38.6	37.1	21.5	2.1	23.6
Harari	21.4	32.6	31.9	33.6	36.6	10.5	4.7	15.2
Addis Ababa	37.2	45.8	60.2	53.0	56.1	23.0	−4.1	18.9
Dire Dawa	26.5	33.7	30.9	31.1	35.4	4.4	4.5	8.9
Place of residence								
Urban	33.1	43.0	53.2	54.5	53.2	20.1	0.0	20.1
Rural	3.7	12.2	24.6	36.3	42.9	20.8	18.3	39.2
Ethiopia	7.2	15.5	29.6	39.2	45.7	22.4	16.1	38.5

Note: FP: family planning; SNNPR, southern nations, Nationalities, and Peoples’ Region; Other religion: Catholic, traditional religions, and other unspecified religions.

### Individual- and Community-Level Factors Influencing Modern Contraceptive Use Among Married and Non-Pregnant Women of Reproductive Age

#### Model Assessment

The AIC value for each survey year was smallest in the fourth model (Model IV/combined model), which incorporated both individual- and community-level factors. Consequently, we chose the combined model, which includes individual- and community-level factors, for our analysis of women’s modern contraceptive use (Supplementary Table S1).

#### Individual-Level Variables

The analysis revealed that in most of the survey years modern contraceptive use is significantly determined by the individual-level factors which include woman’s age, education level, religion, wealth status, total number of living children, birth in the past 3 years, exposure to family planning messages through at least one media outlet, visit to a health facility in the last 12 months, the husband’s desire for children, and the woman’s employment status (Table 3). The results indicated that married and non-pregnant women in the age category of 35–49 had lower odds of using modern contraceptives compared to those in the reference age category of 15–24. For instance, in 2011, those in the age group of 35–49 had an AOR of 0.3 (95% CI: 0.3–0.4) for using modern contraceptives compared to those in the age group of 15–24. *Compared to women with no formal education, those with higher levels of education had greater odds of using modern contraceptives. For example, in 2019, women who completed primary education were 1.7 times more likely to use modern contraceptives (95% CI: 1.5, 2.0). Similarly, women with secondary education had 2.1 times higher odds (95% CI: 1.6, 2.8), and those with higher education had 3.3 times higher odds (95% CI: 2.2, 5.0) of using modern contraceptives. In 2000, women who had completed higher education had 5.6 times higher odds (95% CI: 2.5, 12.3) of using modern contraceptives compared to those with no education.*

**TABLE 3:** Multilevel mixed effect logistic regression analysis of individual and community-level factors associated with modern contraceptive use among married and non-pregnant women of reproductive age in Ethiopia, 2000, 2005, 2011, 2016 and 2019.

Characteristics fixed effect	2000 Model IV AOR (95%CI)		2005 Model IV AOR (95%CI)		2011 Model IV AOR (95%CI)		2016 Model IV AOR (95%CI)		2019 Model IV AOR (95%CI)	
Age Category										
15–24	1		1		1		1		1	
25–34	1.3	(1.0,1.7)	0.9	(0.7,1.1)	0.8	(0.7,0.9)***	0.8	(0.7,1.0)*	0.7	(0.6,0.9)**
35–49	0.6	(0.4,0.9)*	0.5	(0.4,0.6)***	0.3	(0.3,0.4)***	0.4	(0.4,0.5)***	0.3	(0.3,0.4)***
Women’s education level										
No education	1		1		1		1		1	
Primary	1.9	(1.4,2.5)***	1.4	(1.2,1.7)***	1.2	(1.1,1.4)**	1.1	(1.0,1.3)*	1.7	(1.5,2.0)***
Secondary	2.5	(1.7,3.6)***	1.7	(1.2,2.3)**	1.6	(1.2,2.3)**	1.6	(1.2,2.0)***	2.1	(1.6,2.8)***
Higher	5.6	(2.5,12.3)***	1.1	(0.6,2.1)	1.7	(1.2,2.5)**	1.2	(0.9,1.6)	3.3	(2.2,5.0)***
Religion										
Orthodox	1		1		1		1		1	
Protestant	0.7	(0.5,1.0)*	0.8	(0.6,1.0)*	1.1	(0.9,1.3)	1.1	(0.9,1.3)	0.9	(0.8,1.2)
Muslim	1.5	(1.1,2.1)*	0.7	(0.5,0.9)*	0.9	(0.8,1.2)	0.6	(0.5,0.8)***	1.1	(0.8,1.5)
Other religion	1.1	(0.6,2.0)	0.6	(0.4,1.0)*	0.7	(0.5,1.1)	0.6	(0.4,0.9)**	1.3	(0.8,2.1)
Wealth Status										
Poor	1		1		1		1		1	
Middle	1.2	(0.9,1.5)	1.7	(1.4,2.2)***	1.2	(1.0,1.4)*	1.2	(1.1,1.4)**	1.4	(1.1,1.6)**
Rich	1.5	(1.2,1.9)***	2.0	(1.6,2.5)***	1.6	(1.3,1.8)***	1.2	(1.1,1.4)**	1.2	(1.0,1.4).
Total number of children										
None(no child)	1		1		1		1		1	
1–4	3.0	(1.9,4.8)***	4.7	(3.2,6.8)***	3.6	(2.8,4.5)***	2.5	(2.0,3.1)***	5.3	(4.0,6.9)***
5–8	6.1	(3.6,10.3)***	7.0	(4.6,10.6)***	3.9	(3.0,5.2)***	2.2	(1.7,2.8)***	4.8	(3.5,6.5)***
>=9	6.5	(2.9,14.8)***	10.6	(6.2,18.0)***	4.4	(2.9,6.7)***	1.0	(0.7,1.6)	4.2	(2.6,6.9)***
Experience of terminated pregnancy										
No	1		1		1		1		1	
Yes	0.9	(0.7,1.2)	1.0	(0.8,1.3)	0.6	(0.5,0.8)***	0.6	(0.5,0.7)***	0.9	(0.8,1.0)
Birth in the past 3 years category										
No birth	1		1		1		1		1	
one child	0.5	(0.4,0.7)***	0.5	(0.4,0.5)***	0.5	(0.4,0.6)***	0.6	(0.5,0.7)***	0.6	(0.4,0.8)**
>=2 children	0.4	(0.2,0.6)***			0.3	(0.2,0.4)***	0.3	(0.3,0.4)***		
Heard FP message via media										
No	1		1		1		1		1	
Yes	1.4	(1.1,1.8)*	1.2	(1.0,1.4).	1.1	(1.0,1.3)*	1.0	(0.9,1.1)	NA	
Visited health facility in the last 12 months										
No	1		1		1		1		1	
Yes	3.0	(2.4,3.8)***	2.4	(2.1,2.7)***	1.6	(1.5,1.8)***	1.4	(1.2,1.5)***	NA	

Characteristics fixed effect	2000 Model IV AOR (95%CI)		2005 Model IV AOR (95%CI)		2011 Model IV AOR (95%CI)		2016 Model IV AOR (95%CI)		2019 Model IV AOR (95%CI)	
Husbands desire for children										
Both want same	1		1		1		1		NA	
Husband wants more	0.8	(0.6,1.0).	0.8	(0.6,1.0)*	0.6	(0.5,0.7)***	0.7	(0.7,0.8)***	NA	
Husband wants fewer	0.7	(0.5,1.0).	1.1	(0.8,1.5)	0.8	(0.7,1.0)*	0.9	(0.7,1.0).	NA	
Don’t Know	0.5	(0.4,0.7)***	0.6	(0.5,0.7)***	0.4	(0.4,0.5)***	0.7	(0.7,0.8)***	NA	
Partner’s Education										
No education	1		1		1		1		NA	
Primary	1.3	(1.0,1.7).	1.5	(1.3,1.8)***	1.1	(1.0,1.2)	1.2	(1.1,1.4)**	NA	
Secondary	1.3	(0.9,1.8)	1.5	(1.2,1.9)***	0.9	(0.7,1.2)	1.1	(0.9,1.3)	NA	
Higher	1.8	(1.1,2.9)*	1.6	(1.0,2.6)*	0.9	(0.6,1.2)	1.0	(0.8,1.3)	NA	
Don’t know	0.0	(0.0,Inf)	0.0	(0.0,Inf)	2.3	(1.3,4.3)**	0.8	(0.5,1.4)	NA	
Women work status										
No	1		1		1		1		NA	
Yes	1.2	(0.9,1.4)	1.3	(1.1,1.6)***	1.3	(1.2,1.5)***	1.1	(1.0,1.2).	NA	
Region										
Tigray	1		1		1		1		1	
Afar	1.7	(0.7,3.8)	0.5	(0.2,1.3)	0.8	(0.4,1.7)	0.5	(0.2,1.0)*	0.5	(0.0,1.3E+24)
Amhara	1.5	(1.0,2.2).	1.0	(0.8,1.4)	2.6	(2.1,3.4)***	2.0	(1.6,2.5)***	2.3	(1.7,3.0)***
Oromia	0.6	(0.4,0.9)*	0.8	(0.6,1.1)	1.6	(1.2,2.0)***	1.1	(0.8,1.3)	1.8	(1.3,2.4)***
Somali	0.0	(0.0,3.3)	0.3	(0.2,0.7)**	0.2	(0.1,0.5)***	0.1	(0.0,0.2)***	0.2	(0.1,0.4)***
Ben-Gumz	1.7	(0.7,3.7)	0.2	(0.0,0.6)**	1.7	(1.0,2.8).	0.9	(0.6,1.5)	1.4	(0.0,1.6E+12)
SNNPR	0.9	(0.6,1.4)	0.8	(0.5,1.1)	1.4	(1.1,1.9)*	1.4	(1.1,1.8)**	1.8	(1.3,2.5)***
Gambela	1.2	(0.3,4.8)	1.4	(0.4,4.4)	1.5	(0.6,3.5)	0.8	(0.3,2.2)	0.6	(0.0,1.8E+31)
Harari	1.3	(0.3,5.0)	0.6	(0.2,2.1)	1.5	(0.5,4.0)	1.2	(0.4,4.0)	1.0	(0.0,1.0E+63)
Addis Ababa	1.1	(0.6,1.8)	0.7	(0.4,1.1)	2.3	(1.6,3.3)***	1.2	(0.8,1.6)	1.0	(0.6,1.6)
Dire Dawa	1.4	(0.5,3.8)	3.2	(1.3,7.8)*	1.2	(0.5,3.2)	0.9	(0.5,1.9)	2.4	(0.0,5.1E+25)
Place of residence										
Urban	1		1		1		1		1	
Rural	0.1	(0.1,0.2)***	0.8	(0.6,1.1)	0.8	(0.7,1.0)*	0.9	(0.7,1.1)	0.8	(0.7,1.0)*
Community level work										
Low	1		1		1		1		NA	
High	1.5	(1.2,1.9)**	1.1	(0.9,1.3)	1.2	(1.0,1.4)*	1.2	(1.1,1.4)**	NA	NA
Community level wealth										
Low	1		1		1		1		1	
High	6.3E+06	(0.0,Inf)	1.3	(1.1,1.6)**	1.3	(1.2,1.5)***	1.4	(1.2,1.5)***	1.1	(0.9,1.3)
Community level media access (FP)										
Low	1		1		1		1		NA	
High	0.8	(0.6,1.1)	1.7	(1.3,2.1)***	1.3	(1.1,1.6)**	0.9	(0.7,1.2)	NA	
Community level religion composition										
Low	1		1		1		1		1	
High	1.2	(0.8,1.8)	0.8	(0.6,1.1)	1.2	(1.0,1.5).	1.1	(0.9,1.3)	2.3	(1.7,3.1)***

Note: AOR, adjusted odds ratio; CI, confidence interval; FP, family planning; SNNPR, southern nations, Nationalities, and Peoples’ Region. Signif. codes: <0.001 = ***, 0.001 = **, 0.05 = *, 0.05 = .; Other religion: Catholic, traditional religions, and other unspecified religions.

In the four consecutive survey years of 2005, 2011, 2016, and 2019, women from middle and higher household wealth indices were more likely to use modern contraceptives compared to those from lower wealth indices. For instance, in 2011, women from middle wealth status had 1.2 times higher odds (95% CI: 1.0, 1.4) of using modern contraceptives, while women from rich wealth status had 1.6 times higher odds (95% CI: 1.3, 1.8) compared to those from lower wealth status. In 2016, women who had visited a health facility in the past 12 months were 1.4 times more likely to use modern contraceptives (95% CI: 1.2, 1.5) compared to those who had not visited a health facility. Similarly, in 2016, women whose husbands wanted more children were 0.7 times less likely to use modern contraceptives (95% CI: 0.7, 0.8) compared to those whose husbands desired the same number of children as their wives.

In the same year, women whose husbands desired more children were less likely to use modern contraceptives compared to those whose husbands desired the same number of children, with an AOR of 0.7 (95% CI: 0.7, 0.8). Although data on women’s employment status were unavailable for 2019, this variable was consistently identified as a significant factor influencing the use of modern contraceptives among women of reproductive age in the three preceding survey years, with AORs of 1.3 (95% CI: 1.1, 1.6) in 2005, 1.3 (95% CI: 1.2, 1.5) in 2011, and 1.1 (95% CI: 1.0, 1.2) in 2016. Additionally, in 2011 and 2016, women with a history of terminated pregnancy (abortion) were less likely to use modern contraceptives, with AORs of 0.6 (95% CI: 0.5, 0.8) and 0.6 (95% CI: 0.5, 0.7), respectively.

#### Community-Level Variables

Among community-level factors, the region significantly influenced the use of modern contraceptive methods. Women residing in the Somali region were significantly less likely to use these methods compared to those in the reference region of Tigray across all four consecutive survey years. For instance, in 2019, women living in the Somali region were 0.2 times less likely to use modern contraceptives (95% CI: 0.1, 0.4) compared to women in Tigray.

Place of residence was another significant determinant of modern contraceptive use. Specifically, women living in rural areas were less likely to use modern contraceptives than those in urban areas over the past two decades. For example, in 2011 and 2019, women residing in rural areas were 0.8 times less likely to use modern contraceptives (95% CI: 0.7, 1.1) compared to those in urban areas.

In 2005, 2011, and 2016, women from clusters with higher levels of community-level female employment were more likely to use modern contraceptives compared to those from clusters with lower levels of female employment. The adjusted odds ratios (AORs) for these years were 1.5 (95% CI: 1.2, 1.9) in 2005, 1.2 (95% CI: 1.0, 1.4) in 2011, and 1.2 (95% CI: 1.1, 1.4) in 2016 (Table 3).

### Trends in Modern Contraceptive Use

**FIGURE 2:**
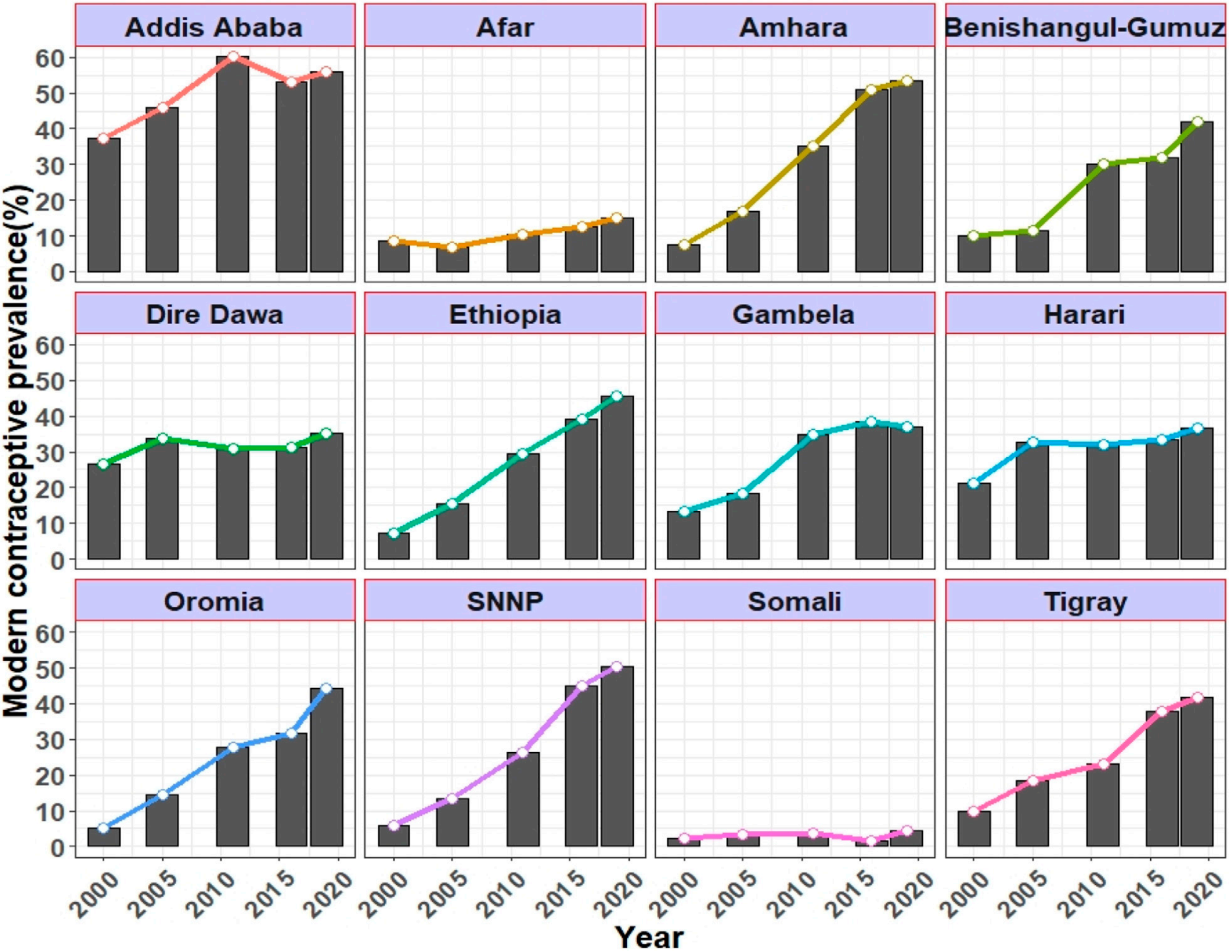
Trends in the use of modern contraceptives by married non-pregnant women (aged between 15–49 years) per region in 2000, 2005, 2011, 2016, and 2019.

The analysis revealed an increase in the percentage of women utilizing modern contraceptive methods over the past two decades (2000–2019). Nationally, the use of modern contraceptives has seen a steady rise, from 7.2% in 2000, to 15.5% in 2005, 29.6% in 2011, 39.2% in 2016, and 45.7% in 2019. To further elucidate the shift in modern contraceptive use, we have broadly divided the trend period into two phases: phase 1 (2000–2011) and phase 2 (2011–2019). The most significant increase was observed in the first decade, with a rise of 22.4%p, compared to the 16.1%p rise in the second decade (Figure 2; Table 2).

Trends in modern contraceptive use varied in terms of women’s characteristics. The greatest increase in modern contraceptive use across age groups was seen among married, non-pregnant women aged 15–24, with an overall rise of 49.4%p during the period from 2000 to 2019. The use of modern contraceptives consistently increased with women’s education levels in each survey year, with the exception of 2005. Examining the regional trend between 2000 and 2019, Amhara, SNNPR, and Oromia ranked first to third with changes of 46.1%p, 44.5%p, and 39.2%p, respectively. While Somali experienced the smallest change 2.0%p followed by Afar region which recorded a 6.5%p change between 2000 and 2019 (Table 2).

## Discussion

In our study, we examined the regional variation and trends and explored the individual- and community-level factors influencing the use of modern contraceptives among married, non-pregnant women of reproductive age in Ethiopia from 2000 to 2019. Our analysis of modern contraceptive use in Ethiopia by region revealed that in 2000 and 2005, Addis Ababa, Dire Dawa, and Harari had the highest usage rates among married, non-pregnant women of reproductive age, ranking first, second, and third respectively. Meanwhile, in 2016 and 2019, the second and third positions were held by the Amhara and SNNP regions respectively. This trend may be attributed to the increased use of modern contraceptives and significant achievements by public health policymakers and implementers in these regions. It may also be linked to the collaborative efforts of government, non-governmental organizations, and other stakeholders in ensuring the accessibility and availability of infrastructure, including reproductive health and family planning services [30–33]. The Somali region, however, had the lowest percentage of women who used modern contraception over time. This low modern contraceptive use may be attributed to the Somali region’s less urbanized and underdeveloped status, compounded by the pastoralist lifestyle of its inhabitants [12]. Not only is the majority of the population in this region relatively less educated, but they also lack knowledge about contraception [34]. Additionally, health facilities in the Somali region are less likely to provide family planning services, covering only 53% [35]. The other pastoralist region, Afar, also showed the lowest percentage point change in modern contraceptives utilization in phase 1(2000–2011) and the total study period (2000–2019). This could be because of the similar reason that is stated for Somali region.

Among the individual-level factors, the household wealth index significantly influenced modern contraceptives use in Ethiopia, which aligns with prior studies [12–16]. This suggests that women with higher income sources are likely to have better access to family planning (FP) services, knowledge, and information. Furthermore, wealthier women, who are highly likely to be involved in employment activities or other business associations, may have a greater desire to limit their fertility. This result is consistent with studies carried out in other countries [17–19, 21, 22]. Studies conducted in Ethiopia had identified that a woman’s educational status influences the use of modern contraceptives, a finding that is in line with our research [13, 14, 16]. Similar results were found in other studies [17–22, 24, 36]. This result suggests that the more educated women become, the more they are likely to use modern contraceptives as their understanding and knowledge of their importance and use increases. Women pursuing higher education often delay pregnancy in order to complete their studies and secure employment. Once employed, the opportunity cost of having a child can motivate them to use modern contraceptives and limit their family size. Our study underscores the importance of empowering women with economic opportunities, enabling them to make voluntary, informed choices and decisions. Exposure to FP messages through multiple media outlets, such as television, radio, and newspapers, was found to positively impact modern contraceptive use among women of reproductive age. This finding is in line with other studies conducted in Ethiopia [14–16], as well as other elsewhere [18, 20, 22, 24]. However, this finding contradicts another research conducted in Ethiopia [12]. This inconsistency may be due to some women’s preference for gaining information through community conversations and social gatherings, rather than through radio, television, or newspapers. This suggests that, within the Ethiopian context, community conversations and individual counseling approaches may be more effective in promoting changes in FP practices among women of reproductive age.

The community-level variable “place of residence,” was identified as a significant determinant of modern contraceptive use, consistent with other research [5, 12–14, 16–19, 21–23]. The explanation for this is that urban women have superior access to contraceptive methods, improved living standards, greater autonomy, and more decision-making power compared to their rural counterparts. Furthermore, urban areas typically have superior infrastructure, including health facilities, and offer more employment opportunities for residents than rural areas. This suggests that urbanization can significantly influence FP practices in Ethiopia. Another community-level variable, region, was identified as a key influential factor affecting the use of modern contraceptives in Ethiopia, a finding that aligns with other studies [12, 13, 16]. The varying social, economic, cultural, and political conditions and stability within regions determine the disparity in modern contraceptive use across different regions.

In the first decade of the study period (2000–2011), the most significant rise in modern contraceptive use was observed in Amhara, Addis Ababa, and Oromia, with increases of 27.6%p, 23.0%p, and 22.6%p respectively. The smallest change, an increase of 1.4%p, was recorded in Somali. Our results contradict those of another study conducted in Ethiopia [37]. That study found that Gambela, Amhara, and SNNPR had the highest increases in the period from 2000 to 2011, with increases of 35.7%p, 33.8%p, and 32.0%p respectively [36]. The discrepancy between the two studies could be due to the fact that the latter study focused on young married women aged 15–24, while our study considered married women aged 15–49. Women of reproductive age who fall into the 15–24 age group are typically more sexually active and are more likely to experiment during this period [38].

In Ethiopia, over the past two decades, there has been a consistent rise in the use of modern contraceptives. This trend aligns with findings from other studies conducted during the same period [1, 11]. This rise may result from the significant focus and effort the Ethiopian government has placed on health policy, particularly family planning (FP), to improve maternal and child health and reduce morbidity and mortality. Especially, *the Ethiopian Health Extension Program, a community-based health service delivery program implemented since 2003, has played a pivotal role in improving public health outcomes, particularly in family planning and the use of modern contraceptives. Through the efforts of Health Extension Workers (HEWs), the program has facilitated access to contraceptive services at the community level, especially in rural and hard-to-reach areas. The HEP’s focus on community-level engagement, health education, and service delivery has likely contributed to the observed increase in modern contraceptive use over time. While the impact of the HEP was not directly analyzed in this study, its potential role in shaping contraceptive use patterns in Ethiopia is acknowledged as a key contextual factor*. Additionally, the involvement of health workers in health education, as well as their participation in various community and institution-based reproductive health services, may have contributed to the positive impact on the rise in modern contraceptive use [39]. However, at the national level in comparison to the first decade modern contraceptive utilization declined in the second decade by 6.3 Percentage point. T*he reduction in modern contraceptive use from the first decade (22.4) to the second decade (16.1) may be attributed to the initial high unmet need for modern contraceptives, which led to substantial increases in utilization during the first decade. As the unmet need declined and contraceptive coverage expanded, subsequent increases became more gradual, reflecting a saturation effect.*

This study has several limitations. Firstly, the use of a cross-sectional EDHS survey dataset prevents us from establishing causal relationships between the explanatory variables and the outcome of interest. Secondly, as the 2019 EDHS was conducted over 5 years ago, our findings may not fully reflect recent changes in contraceptive use behavior. Events such as the COVID-19 pandemic, the 2021 northern crisis in Ethiopia, and the establishment of three new regions from the SNNPR may have significantly influenced contraceptive use among Ethiopian women. Additionally, data for the newly established regions of Sidama and South West Ethiopia Peoples’ Region were not available separately and were treated as part of the former SNNPR. Thirdly, while our study attempted to incorporate both individual- and community-level factors, the list of factors is not exhaustive. Factors such as women’s autonomy, access to health services, and behavioral influences were not accounted for. Furthermore, the reliance on self-reported data introduces the potential for social desirability bias. A potential interaction or effect modification may also exist between women’s visits to health facilities and their husbands’ fertility preferences. For example, women who visit health facilities are more likely to use contraceptives, while those whose husbands desire more children may be less likely to use them. However, this interaction was not explicitly tested in our model. Future research could explore such interactions to better understand the combined influence of these factors on contraceptive use. Fourthly, the EDHS dataset does not inherently provide a multi-level structure. To address this, we constructed a two-level model by aggregating household-level data within enumeration areas (EAs) to create community-level indicators, following widely accepted methods for multi-level analyses using EDHS data. While incorporating a third level (e.g., region or zone) could provide additional insights, our study objectives and the data structure led us to prioritize a two-level model for simplicity and interpretability. Although Latent Class Analysis (LCA) could have been employed to identify unobserved subgroups, this study focused on examining the relationship between specific explanatory variables and contraceptive use. Future studies may consider using LCA to explore latent subgroups in contraceptive behavior. Lastly*, while we applied a weighted analysis to ensure the representativeness of the estimates and to account for the complex sampling design of the EDHS, we recognize that the accuracy of the weighted analysis depends on the quality and reliability of the auxiliary data used to compute the sampling weights. Since the EDHS is a secondary data source, there is a possibility of errors or omissions in the auxiliary data used for weight calculation. This could introduce bias into our estimates. To mitigate this risk, we adhered to the DHS guidelines on the appropriate use of sampling weights and followed established protocols for data cleaning and quality checks prior to analysis. Nevertheless, this remains a limitation inherent to secondary data analysis, and we recommend cautious interpretation of the weighted estimates*. Notably, in 2011 and 2016, women with a history of terminated pregnancy (abortion) were less likely to use modern contraceptives, as indicated by the negative association observed in our models. This finding suggests that such women may face unique psychological, social, or systemic barriers to contraceptive use. While these factors were not further investigated in our study, they warrant exploration in future research.

### Conclusion

*This study reveals that the use of modern contraceptives is determined by both individual- and community-level factors. At the individual level, key determinants include women’s education and household wealth, while at the community level, place of residence and region are significant factors. These findings underscore the importance of addressing both individual and contextual factors to promote equitable access to modern contraceptives. The observed regional variations suggest that structural and contextual community factors, as well as individual characteristics, play a critical role in shaping contraceptive use*. This result highlights the need for family planning services to be tailored at the individual- and community-levels in order to address the disparities in regional variations of modern contraceptive use.
