# Supplementary material for: Evolving Landscape of Modern Contraceptive Use in Ethiopia: A Two-Decade Analysis
Source: Int J Public Health. 2025 Apr 11;70:1607680. doi: 10.3389/ijph.2025.1607680 (PMC12021618; doi:10.3389/ijph.2025.1607680)
Supplement: Supplementary file 1 [file Table1.docx]

**Supplementary table1. AIC, BIC, and ICC values of the successive models of each survey year.**

| **EDHS** | **Indicators** | **Model I** | **Model II** | **Model III** | **Model IV** |
| --- | --- | --- | --- | --- | --- |
| 2000 | AIC | 3657.9 | 3113.7 | 2989.6 | 2839.5 |
|  | BIC | 3671.8 | 3225.4 | 3185.0 | 3132.7 |
|  | ICC | 12.4 | 4.9 | 0.0 | 11.0 |
| 2005 | AIC | 6170.5 | 5646.9 | 5188.8 | 5150.3 |
|  | BIC | 6184.3 | 5764.3 | 5388.9 | 5454.0 |
|  | ICC | 13.8 | 6.0 | 0.0 | 0.0 |
| 2011 | AIC | 9512.2 | 8950.3 | 8272.5 | 8201.2 |
|  | BIC | 9526.1 | 9069.5 | 8482.9 | 8516.8 |
|  | ICC | 16.6 | 9.4 | 0.0 | 0.0 |
| 2016 | AIC | 10896.2 | 10556.6 | 10030.6 | 9983.6 |
|  | BIC | 10910.0 | 10676.0 | 10242.0 | 10301.0 |
|  | ICC | 22.5 | 15.8 | 0.0 | 0.0 |
| 2019 | AIC | 6508.3 | 6319.6 | 5927.2 | 5872.7 |
|  | BIC | 6521.3 | 6416.9 | 6050.5 | 6080.3 |
|  | ICC | 17.7 | 12.0 | 0.0 | 0.0 |

AIC, Akaike information criterion; BIC, Bayesian information criterion.

ICC, intraclass correlation coefficient
